# Reliance Management in Healthcare for Professional and Elite Athletes: Ethics, Patient-Centered Care, and Governance

**DOI:** 10.3390/healthcare14162473

**Published:** 2026-08-10

**Authors:** Zbigniew Waśkiewicz

**Affiliations:** Institute of Sport Sciences, Jerzy Kukuczka Academy of Physical Education in Katowice, 40-065 Katowice, Poland; z.waskiewicz@awf.katowice.pl

**Keywords:** patient-centered care, healthcare ethics, athlete–physician relationship, clinical governance, shared decision-making, confidentiality, dual loyalty, return-to-play, patient safety, elite sport

## Abstract

Professional and elite sport represents a high-pressure healthcare environment in which standard principles of patient-centered care, confidentiality, informed consent, shared decision-making, and clinical independence are tested by organizational and performance-related pressures. This narrative review examines reliance management in athlete–physician relationships. Reliance is conceptually distinguished from trust: whereas trust is an interpersonal attitude that can be betrayed, reliance denotes the broader dependence of the athlete-patient on the physician, the care process, and the surrounding governance system—a dependence that may persist even where trust is absent. Reliance management is conceptualized as the deliberate, multi-level design and maintenance of communicative, ethical, and governance conditions under which such reliance is well-placed. A structured, non-systematic literature search of PubMed, Scopus, Web of Science, SPORTDiscus, and Google Scholar, supplemented by backward citation tracking, informed a thematic synthesis organized around three dimensions: clinical ethics, patient-centered care, and governance. The synthesis distinguishes empirical findings, professional consensus guidance, ethical and legal analysis, and illustrative cases, and identifies domains in which evidence remains weak. An integrative conceptual model is proposed that links governance antecedents at the dyadic, clinical, organizational, and system levels to mechanisms of well-placed reliance and to outcomes that require empirical evaluation. Proposed safeguards—independent medical oversight, documented return-to-play pathways, formal data-sharing rules, second-opinion access, and contractual protection of physician autonomy—are intended to protect patient-centered care and patient safety; their effectiveness has, however, rarely been evaluated and should be the object of future intervention research.

## 1. Introduction

The athlete–physician relationship in professional and elite sport should be understood not only as a sports medicine relationship but also as a distinctive form of healthcare delivery. Athletes at this level are patients whose clinical decisions are often made under conditions of intense time pressure, public scrutiny, financial significance, contractual dependency, and competing organizational interests. These conditions create a healthcare environment in which ordinary principles of clinical ethics and patient-centered care become unusually difficult to protect [1,2,3,4,5].

In conventional healthcare settings, the physician–patient relationship is grounded in trust, confidentiality, informed consent, clinical independence, and the prioritization of patient welfare. In professional and elite sport, these principles remain binding, but their implementation is complicated by the involvement of coaches, managers, sponsors, media, governing bodies, and sometimes fans. The athlete may be treated as a patient, an employee, a public performer, and an organizational asset at the same time. This multiplicity of roles creates ethical and governance challenges that directly affect quality of care, patient safety, and the legitimacy of medical decision-making [1,4,6,7].

For the purposes of this review, the population of interest is defined broadly as athletes competing in professional and elite sport, including professional league athletes, collegiate athletes in high-performance programs, and adolescent athletes in elite development pathways. These groups share exposure to organizational pressure on medical decision-making, but they differ in employment status, consent conditions, safeguarding obligations, and legal protections; these differences are addressed explicitly in Section 3 and Section 4 and in the Limitations.

Operational definition. *Reliance* is defined here as the athlete-patient’s practical dependence on (a) the treating physician, (b) the clinical care process, and (c) the surrounding organizational and regulatory governance system to protect his or her health interests, even when performance, commercial, or institutional pressures point in another direction. Reliance management denotes the deliberate, multi-level design and maintenance of the communicative, ethical, and governance conditions under which this dependence is well-placed.

The central argument of this review is that reliance management is a patient-centered healthcare issue. It cannot be reduced to the personal qualities of the physician or the athlete’s willingness to trust medical advice. Reliance is shaped by clinical communication, shared decision-making, confidentiality practices, return-to-play protocols, employment arrangements, data governance, and oversight mechanisms. When these elements are misaligned, even clinically competent care can become ethically fragile.

The objectives of this review are: (1) to clarify the conceptual relationships between trust, reliance, trustworthiness, and reliance management, and to specify what an integrative reliance-management perspective adds to existing constructs; (2) to synthesize literature on trust, patient-centered communication, shared decision-making, dual loyalty, confidentiality, and return-to-play decisions in professional and elite sport, distinguishing types and strength of evidence; (3) to identify organizational conditions that strengthen or weaken athlete-centered care; and (4) to propose prioritized safeguards for clinicians, healthcare managers, sport organizations, and policymakers, identified explicitly as candidates for empirical evaluation rather than as measures of established effectiveness.

### Trust, Reliance, Trustworthiness, and Reliance Management: Conceptual Foundations

Because the central concept of this review overlaps with several established constructs, their relationships require explicit clarification. In the philosophical literature, trust is standardly analyzed as a species of reliance enriched by normative expectations: the truster accepts vulnerability and expects goodwill or competence from the trustee, and violated trust is experienced as betrayal rather than mere disappointment [8,9]. Reliance is the broader category: one may rely on a person, a process, or an institution without trusting it—for example, because no realistic alternative exists [9]. Trustworthiness, in turn, is a property of the trustee—an individual clinician or an institution—that makes trust warranted; healthcare scholarship emphasizes that institutions can be designed to be more or less worthy of patients’ trust [10,11]. Empirical healthcare research operationalizes interpersonal trust in physicians and, separately, trust in medical institutions and systems, and links both to satisfaction, adherence, and outcomes [11,12,13,14].

These distinctions matter in sport because the two phenomena can dissociate in both directions. An athlete may rely on a team physician without trusting him or her, simply because club structures, insurance arrangements, or selection consequences leave no practical alternative; conversely, an athlete may trust an individual clinician while distrusting the club’s medical system, for example its information-sharing practices [1,15]. Interventions that target only interpersonal trust cannot address the first situation, and interventions that target only organizational structures cannot fully explain the second. A framework is therefore needed that spans the athlete–physician dyad, the clinical microsystem, the employing organization, and the wider regulatory system, and that treats the alignment between these levels as the object of management.

Reliance management, as used here, is that framework. It is presented not as a wholly new atomic construct but as an integrative conceptual model: it combines established constructs—interpersonal and institutional trust, therapeutic alliance, patient-centered care, shared decision-making, and clinical governance [16]—and adds three elements that none of these constructs supplies alone. First, it takes as its unit of analysis the athlete’s overall dependence on the care system, including dependence that persists without trust. Second, it treats trustworthiness as a designable property of structures (contracts, data rules, oversight), not only a virtue of individuals [10]. Third, it directs attention to cross-level alignment: communicative excellence at the dyadic level can be undermined by organizational incentives, and organizational safeguards can be undermined by weak system-level oversight [17,18]. Table 1 distinguishes the core concepts used in this review—their working definitions, the level at which each primarily operates, an example of application in elite sport, and the role each plays in the proposed framework. Section 3.1 presents the integrated conceptual model within which these concepts are located.

## 2. Methods

### 2.1. Design

This review was conducted as a structured narrative review with a documented search strategy and thematic synthesis. A narrative approach was selected because the topic is multidisciplinary and integrates evidence from sports medicine, healthcare ethics, communication science, legal scholarship, organizational governance, and sociology of sport; the aim was interpretive synthesis and conceptual clarification rather than statistical aggregation [21]. Reporting was guided by the quality criteria for narrative reviews summarized in the SANRA scale (justification of importance, explicit aims, description of the search, referencing, scientific reasoning, and appropriate presentation of data) [22].

### 2.2. Search Strategy

Searches of PubMed, Scopus, Web of Science, SPORTDiscus, and Google Scholar were conducted between 1 September and 15 December 2025 and updated on 10 May 2026 to capture recent consensus and policy documents. Searches combined controlled vocabulary (where available) and free-text terms in the following core string, adapted to the syntax of each database: (“athlete–physician relationship” OR “team physician” OR “sports medicine”) AND (ethics OR “patient-centered care” OR “patient-centred care” OR trust OR “shared decision-making” OR “informed consent” OR “dual loyalty” OR “conflict of interest” OR “return to play” OR confidentiality OR “clinical governance” OR autonomy). Only English-language sources were considered. The restriction to English-language sources reflects practical search capacity and the predominance of English-language reporting in the international sports-medicine and sports-ethics literature. Eligible publication types included peer-reviewed empirical studies, ethical and legal analyses, consensus statements and professional guidance, book chapters, and—for illustrative purposes only—official investigative reports and documentary journalism. Backward citation tracking of included sources was used to identify influential consensus statements, ethical analyses, legal discussions, and case-based reports. Full per-database search parameters—including exact search dates, search fields and syntax, applied filters, database editions searched, and the Google Scholar screening protocol—are reported in Supplementary Table S1.

**Figure 1:**
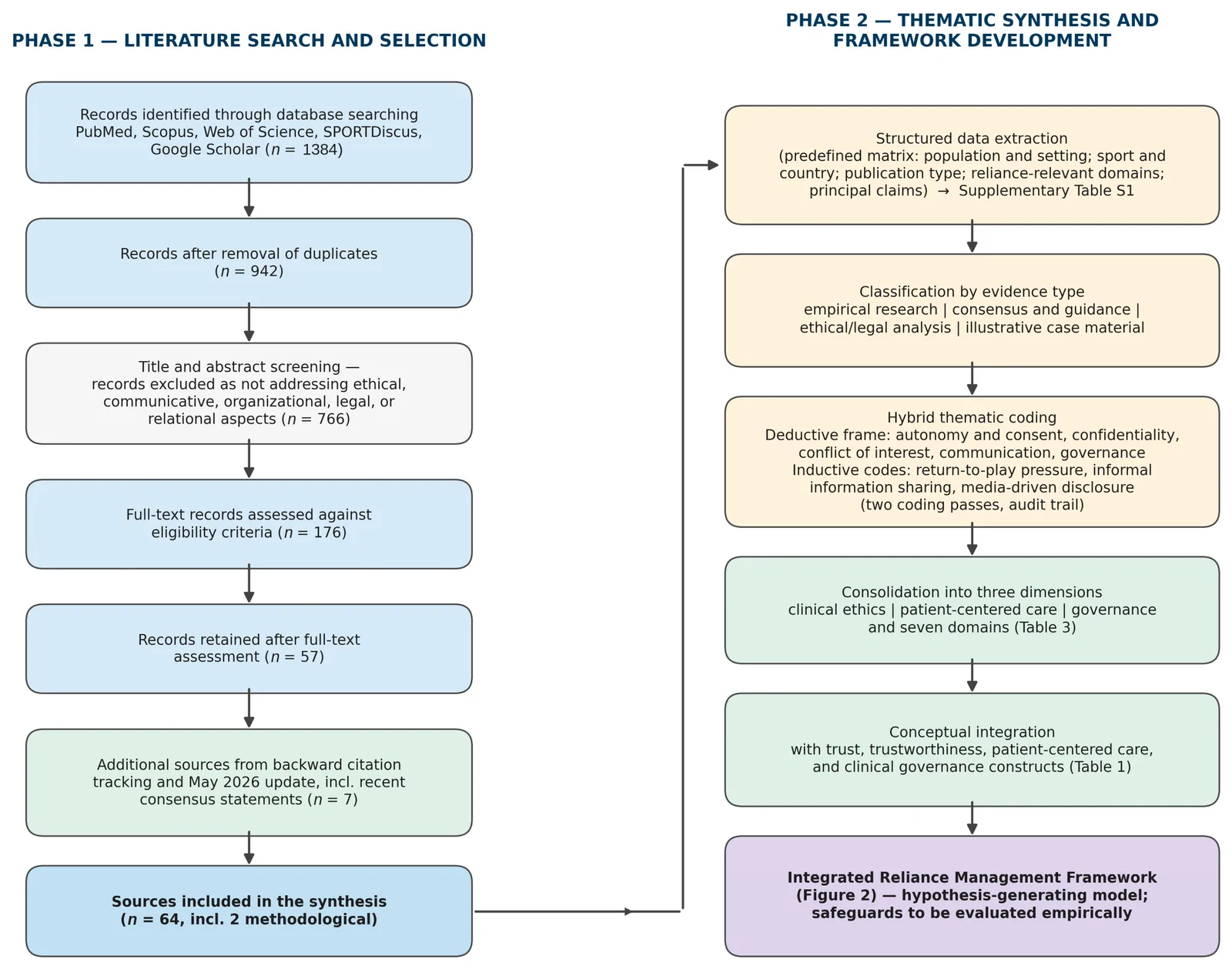
Literature search, thematic synthesis, and development of the reliance management framework. Phase 1 shows the identification and selection of sources; Phase 2 shows the extraction, evidence classification, hybrid thematic coding, and conceptual integration steps through which the framework was developed.

The database searches identified 1384 records, of which 942 remained after de-duplication. Title and abstract screening excluded 766 records that did not address ethical, communicative, organizational, legal, or relational aspects of medical practice in sport or relevant conceptual foundations from general healthcare. Of 176 records assessed in full text, 57 were retained for the synthesis; backward citation tracking and the May 2026 update added a further 7 sources, including recent international consensus statements. Exclusion at the full-text stage followed the eligibility criteria described in Section 2.3 (an ineligible population or setting, absence of a relevant ethical or governance dimension, an exclusively biomedical focus, insufficient conceptual relevance, or an ineligible publication type); however, per-category counts for the 119 excluded full-text records were not retained in a form permitting precise numerical reporting, which we acknowledge as a limitation of reporting granularity (Section 4.3). The final synthesis therefore draws on 64 references, including 2 methodological sources. The flow of records and the subsequent synthesis steps are summarized in Figure 1.

### 2.3. Eligibility and Relevance Judgments

Sources were included when they addressed ethical, communicative, organizational, legal, or relational dimensions of medical practice in professional or elite sport, or when they provided conceptual foundations from general healthcare literature that were directly relevant to the review’s objectives (trust, patient-centered communication, shared decision-making). Sources focused exclusively on biomedical treatment protocols without ethical, communicative, or governance relevance were excluded. Because such criteria are necessarily open, relevance judgments were structured by a predefined extraction matrix recording, for each candidate source: population and setting; sport and country; publication type; the reliance-relevant domain(s) addressed; and the principal claims or findings. General-healthcare sources were retained only when a domain of the emerging synthesis required conceptual or empirical grounding that the sport-specific literature did not supply (for example, the measurement of patient trust and its association with adherence). Evidence from collegiate and adolescent-athlete settings was included as indirect or contextually relevant evidence in domains where directly applicable evidence from employed professional athletes was unavailable (see Section 3.4 and Section 3.5); such evidence is not treated as automatically generalizable to employed professional athletes, and this distinction is flagged explicitly at each point of use. The completed matrix, which also classifies each included source by evidence type, is provided as Supplementary Table S1. The resulting structure of the included literature—the main thematic areas, the number and predominant types of sources in each, key works, and each area’s principal contribution to the framework—is summarized in Table 2.

### 2.4. Thematic Synthesis

The selected literature was analyzed thematically using a hybrid deductive–inductive procedure. An initial deductive frame was derived from established categories of healthcare ethics and patient-centered care (autonomy and consent, confidentiality, conflict of interest, communication, governance). Inductive codes were added where the sport-specific literature raised issues not captured by this frame (for example, return-to-play pressure, informal information-sharing cultures, and media-driven disclosure). Codes were consolidated into the three dimensions that structure the results—clinical ethics, patient-centered care, and governance—and into the seven domains presented in Table 3. Coding was performed by the sole author on two occasions approximately eight weeks apart, and discrepancies between the two passes were resolved by re-reading the source; an audit trail linking each domain to its supporting sources was maintained (Supplementary Table S1). The absence of a second coder is acknowledged as a limitation (Section 6).

### 2.5. Treatment of Evidence

No formal risk-of-bias assessment was conducted, because the purpose of the review was conceptual synthesis rather than systematic evidence grading. The included sources were, however, not treated as equivalent. Each source was classified according to its design and evidentiary role as (a) primary empirical research, (b) evidence synthesis, (c) professional consensus, regulatory, or practice guidance, (d) ethical, legal, conceptual, or policy analysis, (e) illustrative or investigative case material, or (f) a methodological source (Supplementary Table S1). Throughout the Results, statements are qualified according to the class of evidence supporting them, and illustrative case material is used only to exemplify mechanisms, never as proof of prevalence or effectiveness.

## 3. Results: A Healthcare Framework for Reliance Management

### 3.1. Overview of the Framework

The reviewed literature indicates that reliance in athlete–physician relationships is shaped by overlapping clinical, ethical, communicative, and organizational mechanisms. These mechanisms do not operate independently: trust may be strengthened by empathic communication but weakened by conflicts of interest; shared decision-making may be formally present yet substantively limited by organizational pressure; confidentiality may be respected in principle but compromised by informal expectations of information sharing within teams [1,26,29].

**Figure 2:**
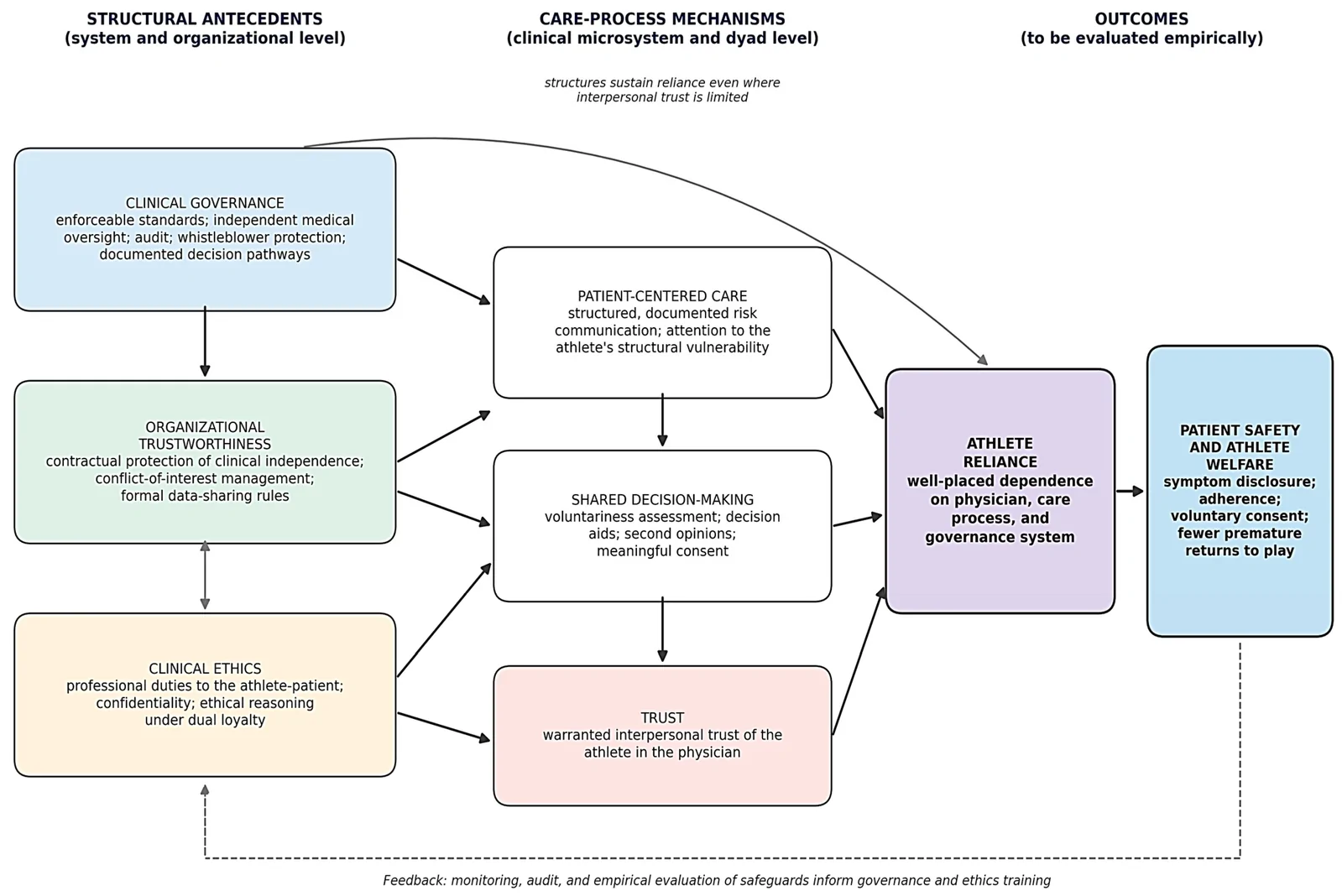
Integrated reliance management framework for athlete–physician relationships. Structural antecedents (clinical governance, organizational trustworthiness, clinical ethics) support care-process mechanisms (patient-centered care, shared decision-making, trust), which together produce well-placed athlete reliance and, in turn, patient safety and athlete welfare. The direct arc from structures to reliance indicates that reliance may be sustained by structures even where interpersonal trust is limited; the dashed arrow indicates evaluative feedback.

The seven domains presented in Table 3 were derived through the coding procedure described in Section 2.4: five emerged directly from recurrent themes in the sport-specific literature (trust and communication; shared decision-making; dual loyalty; return-to-play; confidentiality), and two (organizational culture; system-level oversight) were consolidated from inductive codes concerning institutional and regulatory failures. Figure 2 integrates the results of the synthesis into the proposed conceptual model. It distinguishes structural antecedents at the system and organizational level (clinical governance; organizational trustworthiness; the institutional conditions of clinical ethics), the care-process mechanisms through which they operate at the clinical microsystem and dyad level (patient-centered care; shared decision-making; warranted interpersonal trust), the central construct of well-placed athlete reliance, and the outcomes through which the model should be evaluated—symptom disclosure, adherence, voluntariness of consent, and patient safety. The direct pathway from structures to reliance captures the defining claim of the model: structures can sustain well-placed reliance even where interpersonal trust is limited. The model is explicitly a hypothesis-generating device: the arrows describe proposed, not empirically established, relationships.

The following subsections examine each domain. For each, the nature and consistency of the available evidence is summarized before implications are drawn, and points of disagreement in the literature are identified.

### 3.2. Trust and Patient-Centered Communication

The evidence base for this domain combines a well-developed general-healthcare literature with a small sport-specific one. In general healthcare, interpersonal trust in physicians has been measured with validated instruments and is associated with satisfaction, adherence, and self-reported outcomes in observational studies [11,12,13,14,19]; communication quality is a consistent correlate of these outcomes across reviews in nursing and medicine [20,39,40,41]. Sport-specific evidence is thinner: it includes small qualitative studies of patient trust in athletic training settings [23], surveys of sports medicine physicians’ handling of psychosocial issues [42], and survey evidence linking perceived conflicts of interest to reduced trust and reduced willingness to report concussion symptoms among collegiate American-football players [15]. The mechanisms established in general healthcare are therefore plausible, but mostly unverified, in elite sport.

Within the reliance-management framework, patient-centered communication is not simply a desirable interpersonal skill; it is a protective mechanism. Athletes require clear explanations of diagnosis, prognosis, treatment options, uncertainty, and risk. They also need opportunities to express preferences, fears, career concerns, and doubts without being perceived as disloyal or insufficiently committed to performance. Communication failures may lead to poor adherence, misunderstanding of risk, reduced trust, and ethically weak consent [19,20].

A patient-centered approach requires physicians to recognize that athletes may present as highly autonomous and resilient while still being structurally vulnerable. Contractual dependency, selection pressure, public expectations, and organizational culture may reduce the athlete’s practical freedom to refuse risky options [2,33]. For this reason, communication should be deliberately structured, documented, and repeated when decisions carry major health consequences. This recommendation is normative: no sport-specific trial evidence yet shows that structured communication protects athlete trust or safety.

### 3.3. Shared Decision-Making, Consent, and Athlete Autonomy

Shared decision-making is a well-articulated model in general healthcare [24], and a Cochrane review indicates that interventions can increase its use by professionals, although effects on downstream outcomes are uncertain [43]. In sport, the evidence is largely conceptual and attitudinal. Advocates have argued that return-to-play decisions in elite sport should be shared among athletes, clinicians, and other stakeholders [25], and shared approaches have been described for clinically complex participation decisions such as inherited cardiac rhythm disorders [44]. A survey of healthcare professionals working with athletes found broad endorsement in principle alongside considerable variation in practice [45]. Importantly, the literature is not unanimous: cautionary analyses argue that in elite sport, power asymmetries, competitive incentives, and the risk of shifting responsibility—and potentially liability—onto the athlete can make shared decision-making tokenistic or even ethically problematic if implemented naively [26,46]. This disagreement is substantive, and the present framework sides with neither pole: it treats shared decision-making as valuable but conditional on a decision-making environment that permits meaningful agency.

The main ethical challenge is precisely that shared decision-making may be formally present but substantively weak. An athlete may sign consent or verbally agree to return while experiencing pressure from coaches, teammates, contract negotiations, media narratives, or internalized sporting norms that valorize playing through pain [2,33,47,48]. Under these conditions, voluntariness becomes difficult to assess. The physician’s responsibility is therefore not only to present options, but also to examine whether the decision-making environment allows meaningful agency.

Proposed safeguards include decision aids, written risk summaries, cooling-off periods for high-risk decisions where feasible, independent second opinions, and explicit documentation that the athlete understood both performance benefits and health risks. None of these has been evaluated in elite-sport settings; they are put forward as candidates for implementation research rather than as measures of demonstrated effectiveness.

### 3.4. Dual Loyalty, Clinical Independence, and Conflict-of-Interest Management

Dual loyalty is one of the defining ethical challenges of healthcare in professional sport, and it is the domain with the most developed legal and ethical literature. Team physicians may owe professional duties to the athlete-patient while being employed, contracted, or evaluated by teams, clubs, leagues, or federations; legal scholarship has analyzed the resulting conflicts in detail [4], bioethical analyses have mapped their clinical consequences [6,49,50], and philosophical work has examined the structure of the loyalty conflict itself [27]. Empirical evidence, though limited, is consistent with these analyses: in a survey of collegiate American-football players, athletes who perceived their medical staff as conflicted reported lower trust and greater reluctance to disclose concussion symptoms [15], and interview studies with collegiate athletic trainers document organizational pressure on clinical decisions [51,52].

Notably, the literature disagrees about remedies. Within the same Hastings Center Report series on the National Football League (NFL), one proposal advocates a structural division between a “players’ doctor” and a “club evaluation doctor” [17], while an accompanying analysis questions the feasibility and possible unintended consequences of such separation and favors strengthening professional standards within existing arrangements [53]; a player-physician commentary emphasizes relational and professional safeguards over structural redesign [54]. Analyses of concussion management have likewise reached different conclusions about how far conflict-of-interest management can be achieved without independent physicians [55]. These unresolved disagreements matter for practice: they imply that the choice among structural remedies is currently a matter of reasoned judgment, not settled evidence.

The problem should not be framed as a failure of individual morality alone. Even highly ethical clinicians can be placed in structurally compromised positions when employment arrangements, information flows, or organizational expectations create pressure to align clinical judgment with institutional goals [17,52]. Reliance management therefore requires governance mechanisms that make clinical independence possible in practice: explicit contractual clauses protecting physician autonomy, mandatory disclosure of conflicts of interest, separation between treating and evaluative roles where feasible, independent review for contested return-to-play decisions, and mechanisms allowing athletes to seek second opinions without penalty. As with the previous domain, these are normative proposals whose effectiveness remains to be evaluated.

### 3.5. Return-to-Play Decisions as Patient Safety Decisions

Return-to-play decisions are often presented as routine sports medicine decisions, but they should also be understood as patient safety decisions. The available evidence is of three kinds. First, professional consensus statements—from the early team-physician consensus [3] through its recent musculoskeletal update [56] and the Amsterdam international consensus on concussion in sport [28]—specify medically grounded, graduated return pathways; these documents represent expert agreement rather than trial evidence. Second, empirical studies document pressure on clinicians: in a survey of collegiate sports medicine clinicians, a substantial minority reported pressure from coaches or athletes to prematurely clear concussed athletes [29], and qualitative studies of collegiate athletic trainers describe organizational conflict around clearance decisions [51,52]. It should be noted that this empirical evidence comes chiefly from United States collegiate settings, and its transferability to professional leagues—where employment relations differ—has not been established. Third, ethical analyses frame return-to-play as a risk-communication and consent problem [46,57].

A further genuine controversy concerns the locus of responsibility: it has been argued that return-to-play decisions should not rest with the team physician alone, precisely because of conflicting pressures, and that responsibility should be shared or externalized [30,58]; others emphasize retaining physician authority while strengthening its protections [3,56]. The reliance problem becomes visible when guideline-based care conflicts with organizational expectations: if athletes believe medical decisions are influenced by team performance, trust deteriorates [15]; if physicians believe conservative decisions may be questioned by management, clinical independence may be weakened [29,52]; if return decisions are poorly documented, accountability becomes difficult.

A patient safety approach requires standardized protocols, conservative thresholds for high-risk conditions such as concussion, multidisciplinary clearance, and transparent documentation of risk–benefit reasoning [28,56]. These procedures do not eliminate uncertainty, but they are intended to reduce the likelihood that uncertainty is resolved in favor of organizational convenience rather than athlete welfare. Whether they achieve this has not been directly evaluated.

### 3.6. Confidentiality and Medical Data Governance

Confidentiality is a foundational component of healthcare ethics, yet professional sport creates unusual pressure on medical information. Injury status, prognosis, medication use, psychological readiness, and rehabilitation progress may have competitive, financial, contractual, and media significance, making athlete health data both clinically sensitive and organizationally valuable [1,6,49]. The empirical evidence here is qualitative and partly dated: the most direct study—interviews in English professional football—documented routine informal sharing of medical information with coaching staff and normalized breaches of confidentiality [1]; more recent survey material from player unions indicates that confidentiality concerns persist internationally, though such reports rely on self-selected samples [31]. Mental-health information raises these stakes further, as reflected in international consensus guidance on athlete mental health [59].

Regulatory context matters and varies. Data-protection law in the European Union, health-privacy law in the United States, and sport-specific instruments such as the World Anti-Doping Agency’s International Standard for the Protection of Privacy and Personal Information impose different obligations on clubs, leagues, and anti-doping organizations [32]. What counts as lawful and legitimate sharing of athlete health data therefore differs across jurisdictions—a point developed in Section 4.3.

Reliance is undermined when athletes are uncertain about who receives medical information, what is disclosed, and for what purpose. Even when disclosure is technically authorized, consent may be ethically fragile if athletes feel unable to refuse [1,46]. The protection of confidentiality in sport therefore requires more than informal professionalism; it requires formal data governance: defined access rights, distinction between clinical and performance data, written consent for disclosure, documentation of data-sharing decisions, and clear explanations to athletes of confidentiality boundaries. These governance proposals are consistent with regulatory standards but their effects on athlete trust and disclosure have not been studied.

### 3.7. Organizational Culture, Governance, and System-Level Oversight

Sociological and qualitative research documents cultures of “playing hurt” in which competing while injured is normalized and rewarded [2,33,47,48]; occupational-safety analyses reach similar conclusions about risk normalization in professional sport [34]. Ethical analyses of sports medicine practice argue that such cultures place clinicians under chronic countervailing pressure [5,7,35,60,61].

At the system level, the evidence consists mainly of illustrative case material from official investigations and documentary journalism, which must be interpreted with care: cases come to light selectively, and generalizing from scandals risks overstating prevalence. With that caveat, the cases are instructive about mechanisms. Investigative reporting and subsequent litigation on concussion in the NFL illustrate how institutional interests can delay recognition of long-term harm [62,63]; the United States Anti-Doping Agency’s reasoned decision in the US Postal Service cycling case and the McLaren Report on state-sponsored doping illustrate failures of medical and governance oversight on an organizational and national scale [37,64]; analyses of physicians’ roles in doping describe how clinicians can drift from assistance to negligence where oversight is weak [18]. International consensus guidance on safeguarding athletes from harassment and abuse underscores that welfare failures are typically system failures, not only individual ones [36].

A governance perspective shifts attention from the private clinical encounter to the institutional conditions that make ethical care sustainable. Independent medical committees, external audits, whistleblower protections, athlete advocacy structures, and enforceable standards are proposed to strengthen reliance by reducing dependence on informal goodwill [17,18,36]. These mechanisms are particularly important where athletes are young, financially vulnerable, injured, or dependent on selection decisions [36,48]. Governance cannot, however, replace professional judgment; its role is to create conditions under which professional judgment can be exercised independently, transparently, and consistently with patient-centered care. The interaction between governance and individual professionalism is examined further in Section 4.2.

## 4. Discussion

### 4.1. Principal Findings

This review reframes reliance management in athlete–physician relationships as a problem of healthcare ethics, patient-centered care, and governance. The central conclusion is that reliance is not reducible to interpersonal trust. It emerges from the alignment, or misalignment, between clinical communication, ethical duties, organizational structures, and oversight mechanisms. The conceptual analysis in Section Trust, Reliance, Trustworthiness, and Reliance Management: Conceptual Foundations gives this claim more precision than the trust literature alone can: because athletes may rely without trusting, and may trust individuals while distrusting systems, interventions confined to one level cannot secure well-placed reliance.

Many recommendations in sports medicine focus on the behavior of individual clinicians: communicate better, disclose risk, respect autonomy, maintain confidentiality. These recommendations remain necessary, but the synthesized evidence—clinician-pressure surveys, athlete-trust surveys, and qualitative studies of information-sharing cultures [1,15,29,51]—indicates that they are insufficient when the clinical environment itself weakens patient-centered care. A physician cannot fully protect athlete welfare if confidentiality rules are ambiguous, if employment structures compromise independence, or if return-to-play decisions are shaped by unmanaged conflicts of interest.

### 4.2. Governance and Individual Ethics as Complementary Mechanisms

The governance emphasis of this review should not be read as implying that structural reform alone can protect athlete welfare. Governance structures and individual professional ethics operate as complementary and mutually dependent mechanisms. Structures without professionalism produce compliance theater: formally independent physicians can still be captured by informal loyalties, and consent forms can be signed without understanding. Professionalism without structures produces heroic but fragile ethics: individual clinicians bear the full weight of resisting organizational pressure, a burden the qualitative literature shows to be unrealistic in the long run [29,51,52]. The reliance-management model in Figure 2 therefore places interpersonal skills (communication, shared decision-making) and structural safeguards (contracts, data rules, oversight) at different levels of the same system, connected by bidirectional arrows: good structures make ethical practice ordinary rather than heroic, and ethical practitioners are needed to operate structures in the spirit intended [7,10]. Educational implications follow: ethics and communication training for sports medicine clinicians should address institutional pressure explicitly, not only dyadic technique [35,61].

### 4.3. International and Cross-System Applicability

Most of the reviewed evidence originates from North American professional and collegiate sport and from European professional football. The framework’s components are therefore not uniformly applicable across countries and legal systems, and four sources of variation deserve emphasis. First, physician independence has different institutional meanings where team physicians are club employees, self-employed contractors, federation appointees, or public-health-system clinicians. Second, confidentiality regulation differs materially between, for example, European Union data-protection law, United States health-privacy and labor law, and sport-specific privacy standards [32]; identical data-sharing practices may be lawful in one jurisdiction and unlawful in another. Third, employment structures range from collectively bargained league systems with player unions [17,31] to individual-contract systems with weak athlete representation, which changes the feasibility of contractual safeguards. Fourth, athlete rights and safeguarding obligations—particularly for minors—are codified to very different degrees across countries [36]. The framework should therefore be read as a structured menu of safeguards whose priority, legal form, and feasibility must be adapted to the governance context of each sport and jurisdiction, rather than as a universal prescription.

### 4.4. Populations and the Boundaries of the Evidence

A related caution concerns populations. Several key empirical studies concern collegiate athletes [15,29,51,52] or adolescent elite athletes [48], whereas the review’s primary focus is professional and elite sport. These groups are not interchangeable: collegiate athletes in the United States have historically not been employees, adolescent athletes cannot provide autonomous consent in the way adults can, and safeguarding obligations differ accordingly [36]. The direction of the core findings—organizational pressure on clinical decisions, trust erosion under perceived conflicts of interest—is consistent across these settings, which supports the framework’s plausibility, but effect sizes and mechanisms may differ, and professional-league replications are largely lacking. Where this review draws on collegiate or adolescent evidence, this is indicated explicitly in Section 3.

### 4.5. Transferability Beyond Sport

Professional sport illustrates a broader healthcare problem: clinical relationships embedded in institutional settings where third parties hold strong interests in the patient’s health status, functional capacity, or return to work. Analogous dual-loyalty structures have been described in occupational health, military medicine, and detention settings [38]. It is plausible that elements of the reliance-management framework—independent oversight, formal data-sharing rules, protected second opinions—could transfer to such settings, but this review has not examined their studies systematically. The claim of transferability is therefore presented as a hypothesis for future comparative research rather than as a conclusion of this synthesis.

### 4.6. What the Present Synthesis Can and Cannot Establish

The case-based material discussed in Section 3.7 shows that reliance can collapse when patient welfare is subordinated to institutional goals, and it exposes recurring vulnerabilities: unclear accountability, conflicts of interest, weak data boundaries, insufficient independence, and inadequate protection for those who raise concerns [37,63,64]. What the present evidence base cannot establish is the effectiveness of the proposed remedies. The safeguards assembled in Table 3 are grounded in ethical reasoning, professional consensus, and regulatory standards; almost none has been evaluated in intervention or implementation studies. The appropriate conclusion is therefore conditional: these measures are intended to protect patient-centered care and patient safety, they are coherent with the available observational evidence, and they should be implemented in ways that permit evaluation.

## 5. Practical Implications

The implications of this review are presented in three tiers, ordered by the strength of their ethical rationale, their proximity to patient safety, and their feasibility. This prioritization is itself a judgment of the author based on the synthesis, not an empirically derived ranking.

1. Essential safeguards (patient-safety floor): Three measures follow most directly from the convergence of ethical analysis, consensus guidance, and observational evidence, and should be regarded as the minimum standard: (1) explicit contractual and policy protection of physician clinical independence, including disclosure of conflicts of interest [4,17,53]; (2) standardized, documented, and auditable return-to-play pathways for high-risk conditions, above all concussion [28,56]; and (3) written, athlete-comprehensible rules governing medical confidentiality and data sharing, aligned with applicable data-protection and anti-doping privacy standards [1,32].
2. Desirable safeguards (strengthening athlete agency): A second tier strengthens the athlete’s position within care processes: guaranteed access to independent second opinions without selection or contractual penalty [25,30]; separation of treating and evaluative roles where organizational scale permits [17,55]; and integration of ethics and communication training—explicitly addressing coercion, voluntariness, dual loyalty, and communication under pressure—into sports medicine education [35,45,61].
3. Long-term policy development: The third tier requires action by leagues, federations, and regulators over longer time horizons: external oversight mechanisms capable of reviewing systemic failures rather than relying on internal organizational accountability [18,36,37]; enforceable minimum standards for athlete healthcare governance in league and federation regulations; and—critically—commissioned evaluation of all of the above, so that the safeguards’ presumed benefits for trust, disclosure, and patient safety are tested rather than assumed.

## 6. Limitations

This review has several limitations. First, its narrative design means that the synthesis is interpretive rather than exhaustive, and no formal risk-of-bias assessment was conducted; although sources were classified by evidence type (Supplementary Table S1), the weighting of sources within the synthesis reflects the author’s judgment. Second, all screening, selection, and thematic coding were performed by a single author. Repeated coding at two time points and an audit trail were used to improve consistency, but the absence of dual screening and coding creates a risk of selection and interpretive bias that these measures can reduce but not eliminate. Third, the open inclusion criteria appropriate to a conceptual review create a related risk of publication-selection bias: sources consistent with the emerging framework may have been more salient during searching and screening than disconfirming material.

Fourth, the evidence base mixes athlete populations. Several central empirical studies concern United States collegiate athletes or adolescent elite athletes rather than professional athletes, and their findings may not transfer across employment and consent contexts (Section 4.4). Fifth, much of the literature is concentrated in North American and European sport, particularly American football, association football, and collegiate athletics; the framework’s applicability to other regions, sports, and healthcare systems is correspondingly uncertain (Section 4.3). Sixth, restricting inclusion to English-language sources may have introduced language bias: given that healthcare governance, confidentiality regulation, and legal protections for athletes vary substantially across countries, relevant evidence published in other languages—particularly concerning non-Anglophone sporting and healthcare systems—may have been omitted. Seventh, the framework was developed from published literature without direct involvement of athletes, team physicians, or other stakeholders; for a framework whose subject is patient-centeredness, the absence of patient input is a substantive limitation, not merely a procedural one. Eighth, the effectiveness of the proposed safeguards has, with few exceptions, not been empirically evaluated: Table 3 should be read as a set of hypotheses. Finally, reliance management remains an emerging concept; the model in Figure 2 is a basis for theoretical refinement and empirical testing rather than a validated framework.

## 7. Future Directions

Future research should move from conceptual argument to empirical evaluation along five specific lines. First, measurement: a reliance-management or athlete-reliance scale should be developed and validated—plausibly adapting existing physician-trust and organizational-trust instruments [11,12]—with parallel athlete and clinician versions, so that levels of warranted reliance can be quantified and compared across settings. Second, athlete experience: qualitative studies should examine how athletes—including women athletes, athletes in lower leagues, para-athletes, and athletes outside North America and Western Europe—experience confidentiality, consent, return-to-play pressure, and second-opinion access; co-designed studies involving athlete representatives would also address the stakeholder gap identified in Section 6. Third, comparative governance research: the same safeguards should be studied across leagues, sports, and countries with different employment and regulatory structures, to identify which governance arrangements are associated with higher athlete trust and disclosure. Fourth, intervention and implementation studies: specific safeguards—independent review of contested return-to-play decisions, formal data-access rules, guaranteed second opinions—should be introduced in ways that permit controlled or quasi-experimental evaluation against outcomes such as symptom disclosure, adherence, perceived voluntariness, and re-injury rates. Fifth, longitudinal research should track how changes in employment structures and physician-independence protections relate to athlete trust and patient-safety outcomes over time.

## 8. Conclusions

Reliance management in athlete–physician relationships is best understood as a healthcare ethics and governance challenge. Trust and communication remain essential, but they are not sufficient when organizational pressures, conflicts of interest, weak confidentiality rules, or ambiguous accountability structures compromise patient-centered care. Professional and elite athletes are patients in a distinctive clinical environment, and protecting their welfare requires systems that support clinical independence, meaningful consent, transparent risk communication, data protection, and accountable oversight.

The framework proposed here integrates established concepts—trust, trustworthiness, patient-centered care, shared decision-making, and clinical governance—into a multi-level model of well-placed reliance. Its safeguards are intended to strengthen quality of care and patient safety in professional and elite sport; whether they do so is an empirical question that the research agenda in Section 7 is designed to answer. If validated, the model may also inform other healthcare settings shaped by powerful third-party interests, a possibility this review identifies as a hypothesis rather than a finding.

## Figures and Tables

**Table 1:** Conceptual distinction between trust, reliance, trustworthiness, patient-centered care, clinical governance, and reliance management.

Concept	Working Definition	Primary Level of Operation	Example of Application in Elite Sport	Role in the Proposed Framework
Trust	Attitude of accepted vulnerability combined with normative expectations of the trustee’s goodwill and competence; violation is experienced as betrayal [8,11]	Interpersonal (athlete–physician dyad); analogues at institutional level	An athlete follows rehabilitation advice because she is confident the physician acts in her long-term interest	Proximal relational mechanism, produced by trustworthy care processes (Section 3.1)
Reliance	Practical dependence on a person, process, or system to perform as expected; possible without trust (e.g., in the absence of alternatives); violation is experienced as disappointment [9]	Dyadic, organizational, and system levels simultaneously	An athlete continues to consult the club physician, despite limited trust, because insurance and selection rules leave no realistic alternative	Central object of the model: the dependence to be made well-placed
Trustworthiness	Property of a clinician or institution that makes trust and reliance warranted (competence, honesty, aligned incentives, accountable structures) [10,11]	Individual and institutional	A club publishes—and demonstrably follows—written rules on who may access athletes’ medical data	Designable property that safeguards are intended to build
Patient-centered care	Care organized around the patient’s needs, values, preferences, and understanding, enacted chiefly through communication and shared decisions [19,20]	Clinical encounter (dyad and clinical microsystem).	Structured, documented risk disclosure before a return-to-play decision, repeated when the stakes change.	Care-process mechanism translating structures into experienced care
Clinical governance	Systematic organizational approach through which institutions are accountable for safeguarding standards and continuously improving quality of care [16]	Organizational and system	Independent review of contested clearance decisions; audits of decision documentation.	Structural antecedent creating the conditions for clinical independence
Reliance management	Deliberate, multi-level design and maintenance of communicative, ethical, and governance conditions under which athlete reliance is well-placed	Cross-level (dyad ↔ clinical microsystem ↔ organization ↔ system)	Combined package: contractual independence, documented pathways, data rules, protected second opinions	Integrative model linking the concepts above (this review; Section 3.1)

**Table 2:** Characteristics of the literature included in the narrative synthesis (*n* = 64 sources). Thematic areas correspond to the coding structure described in Section 2.4; the full source-level classification is provided in Supplementary Table S1. Thematic categories are mutually exclusive: each source was assigned to the single predominant thematic domain most closely aligned with its principal contribution to the synthesis, even where secondary relevance to other domains existed; column *n* therefore sums to the total number of included sources (*n* = 64).

Thematic Area	*n*	Predominant Publication Types	Key Works	Main Contribution to the Framework
Trust, reliance, and trustworthiness: concepts and measurement (philosophy; general healthcare)	9	Conceptual and philosophical analyses; measurement studies; surveys	[8,9,10,11]	Conceptual foundation of the trust–reliance distinction; measurement models for future scale development (Section Trust, Reliance, Trustworthiness, and Reliance Management: Conceptual Foundations; Table 1).
Patient-centered care and communication	5	Reviews; qualitative and survey studies	[19,20,23]	Communication as a protective mechanism (Section 3.2).
Shared decision-making and consent	7	Conceptual models; systematic review; surveys; critical analyses	[24,25,26]	Care-process mechanisms and their limits under pressure (Section 3.3).
Dual loyalty and conflicts of interest	10	Legal and ethical analyses; athlete survey; qualitative clinician studies	[4,15,17,27]	Organizational trustworthiness; conflict-of-interest safeguards (Section 3.4).
Return-to-play and patient safety	9	Consensus statements; surveys; qualitative studies; position papers	[3,28,29,30]	Clinical-microsystem safeguards; documented decision pathways (Section 3.5).
Confidentiality and health data governance	4	Qualitative study; organizational report; regulatory standard; consensus statement	[1,31,32]	Formal data-governance rules (Section 3.6).
Organizational culture and playing hurt	5	Sociological and survey studies; occupational-safety review	[2,33,34]	Risk normalization; structural vulnerability of athletes (Section 3.2 and Section 3.7).
Ethics of sports medicine: foundations	5	Monographs; ethical analyses	[7,35]	Clinical-ethics dimension; training implications (Section 3.7 and Section 4.2).
Clinical governance and system-level oversight (incl. doping and safeguarding cases)	7	Policy analysis; ethical analyses; investigative reports; documentary material	[16,18,36,37]	System-level antecedents; illustrative failure mechanisms (Section 3.7).
Dual loyalty beyond sport	1	Professional guidelines/report	[38]	Transferability hypothesis (Section 4.5).
Review methodology	2	Methodological guidance	[21,22]	Design and reporting of the review (Section 2).

**Table 3:** Practical governance safeguards for athlete–physician relationships. The final column indicates the current basis of support for the recommended safeguards; “normative” denotes recommendations grounded in ethical or professional reasoning whose effectiveness has not yet been empirically evaluated.

Domain	Principal Risk in Elite Sport	Recommended Organizational Safeguards	Primarily Responsible Actors	Current Basis of Support
Trust and clinical communication	Athletes may comply without fully understanding risk, especially under time pressure	Structured consultations; documentation of key discussions; plain-language explanations; communication training	Medical staff; clubs (training provision)	General-healthcare evidence for trust–communication–adherence links; sport-specific evidence limited to small studies; safeguards normative.
Shared decision-making and autonomy	Shared decision-making may become tokenistic when the athlete is pressured to return	Decision aids; written risk summaries; cooling-off periods where feasible; access to independent advice	Medical staff; clubs; player associations.	Consensus/conceptual support and professional-survey evidence; aid effectiveness untested in sport.
Dual loyalty and conflicts of interest	Team goals, contracts, media pressure, and financial incentives may influence clinical judgment	Conflict-of-interest disclosure; separation of treatment and evaluation roles; contractual protection of physician independence	Clubs; leagues and federations.	Legal/ethical analyses and one empirical conflict–trust association; structural remedies unevaluated.
Return-to-play and patient safety	Premature return may increase injury recurrence, concussion sequelae, or cumulative harm	Standardized protocols; multidisciplinary clearance; conservative thresholds for high-risk injuries; documented rationale	Medical staff; clubs; leagues (protocol mandates)	Consensus statements; clinician-pressure evidence (chiefly collegiate); protocol effectiveness unevaluated.
Confidentiality and health data governance	Teams and media may seek access to injury status, prognosis, or sensitive medical data	Formal data-sharing rules; restricted access; written consent procedures; audit trails	Clubs; leagues; anti-doping organizations	Qualitative/survey evidence of information-sharing problems; regulatory standards exist; safeguard effectiveness unevaluated.
Organizational culture and healthcare quality	A win-at-all-costs culture may normalize playing hurt and weaken athlete agency	Ethics committees; athlete welfare policies; independent reporting channels; governance audits	Clubs; leagues and federations	Sociological and qualitative evidence on playing-hurt cultures; safeguards normative
System-level oversight	Weak oversight can allow systemic failures in anti-doping, safeguarding, or clinical independence	Independent medical governance; whistleblower protection; enforceable standards; external review	Leagues, federations, regulators, anti-doping and safeguarding bodies	Illustrative case material from investigative reports; international consensus guidance; safeguards normative

## Data Availability

No primary empirical dataset was generated for this study. All data extracted and classified for the narrative synthesis—i.e., the source-level extraction and evidence-classification matrix—are provided in Supplementary Table S1.

## Supplementary Materials

The following supporting information can be downloaded at https://www.mdpi.com/article/10.3390/healthcare14162473/s1, Table S1: Characteristics and evidence classification of the sources included in the synthesis.
