# Supplementary material for: Reliance Management in Healthcare for Professional and Elite Athletes: Ethics, Patient-Centered Care, and Governance
Source: Healthcare (Basel). 2026 Aug 10;14(16):2473. doi: 10.3390/healthcare14162473 (PMC13512438; doi:10.3390/healthcare14162473)
Supplement: Supplementary file 1 [file healthcare-14-02473-s001.zip › healthcare-4449564-supplementary.pdf]

Supplementary Table S1

Table S1. Characteristics and evidence classification of the sources included in the synthesis. Reference numbers correspond to the reference list of the main manuscript. Evidence classes: empirical research (quantitative or qualitative), professional consensus or guidance, ethical/legal/conceptual analysis, illustrative case material (official investigative reports, documentary journalism), regulatory standards, and methodological sources. Illustrative case material was used only to exemplify mechanisms, never as evidence of prevalence or effectiveness.

Ref.	Source	Publication type / evidence class	Setting and population	Contribution to framework domain(s)
1	Waddington & Roderick, 2002	Empirical — qualitative (interviews)	English professional football (clinicians, players)	Confidentiality; organizational culture
2	Roderick et al., 2000	Empirical — qualitative (interviews)	English professional football (players)	Playing-hurt culture; athlete agency
3	Herring et al., 2002	Professional consensus statement	Team physicians (USA)	Return-to-play
4	Calandrillo, 2005	Legal analysis	US professional sport	Dual loyalty; conflicts of interest
5	Tamburrini & Tännsjö (Eds.), 2009	Ethical analysis (edited volume)	Elite sport	Ethics foundations
6	Testoni et al., 2013	Ethical analysis	Sports medicine	Ethics; confidentiality; dual loyalty
7	McNamee, 2014	Ethical analysis (monograph)	Sport and medicine	Ethics; governance
8	Baier, 1986	Conceptual/philosophical analysis	General	Trust vs. reliance distinction
9	Hawley, 2014	Conceptual/philosophical analysis	General	Trust vs. reliance distinction
10	O'Neill, 2002	Conceptual analysis (bioethics monograph)	Healthcare institutions	Trustworthiness of institutions
11	Hall et al., 2001	Conceptual and measurement review	General healthcare (USA)	Trust constructs and measurement
12	Thom et al., 2004	Measurement/empirical review	US healthcare quality assessment	Trust measurement
13	Lee & Lin, 2009	Empirical — quantitative (survey)	Patients with diabetes	Trust–adherence–outcome links
14	Petrocchi et al., 2019	Empirical — quantitative (dyadic analysis)	General practice (patients and physicians)	Trust–communication association
15	Baugh et al., 2020	Empirical — quantitative (survey)	US collegiate football players	Conflicts of interest; trust; symptom disclosure
16	Scally & Donaldson, 1998	Conceptual/policy analysis	NHS England; general healthcare	Clinical governance concept
17	Cohen et al., 2016	Ethical/legal analysis and proposal	NFL (professional football, USA)	Dual-loyalty remedies (structural separation)
18	Dikic et al., 2013	Ethical analysis	Anti-doping; sports physicians	System-level oversight
19	Stewart, 1995	Review of empirical studies	General healthcare	Communication–outcome links
20	Kwame & Petrucka, 2021	Literature review	Nursing/general healthcare	Patient-centered communication
21	Green et al., 2006	Methodological guidance	—	Narrative review methodology
22	Baethge et al., 2019	Methodological instrument (SANRA)	—	Narrative review quality criteria

Ref.	Source	Publication type / evidence class	Setting and population	Contribution to framework domain(s)
23	David & Hitchcock, 2018	Empirical — qualitative (interviews)	US athletic training setting	Athlete-patient trust
24	Elwyn et al., 2012	Conceptual model	General clinical practice	Shared decision-making model
25	Dijkstra et al., 2017	Editorial/conceptual	Elite sport	Shared decision-making in return-to-play
26	Paul et al., 2022	Conceptual/critical analysis	Elite sport	Cautionary view of shared decision-making
27	Hanson, 2018	Philosophical analysis	Team physicians	Structure of dual loyalty
28	Patricios et al., 2023	International consensus statement	Concussion in sport (international)	Return-to-play protocols
29	Kroshus et al., 2015	Empirical — quantitative (survey)	US collegiate sports medicine clinicians	Return-to-play pressure
30	Matheson et al., 2011	Conceptual/position paper	Sports medicine	Locus of return-to-play responsibility
31	FIFPro, 2016	Organizational report (survey-based)	Professional footballers (global)	Employment conditions; confidentiality concerns
32	WADA, 2021	Regulatory standard	Anti-doping (international)	Health-data privacy standards
33	Nixon, 1993	Conceptual/sociological analysis	US sport	Playing-hurt culture
34	Chen et al., 2019	Review (occupational safety and health)	Professional sport	Risk normalization; injury
35	Greenfield & West, 2012	Ethical analysis/review	Sports medicine	Ethical decision-making and training
36	Mountjoy et al., 2016	International consensus statement	Sport safeguarding (international)	Harassment/abuse; safeguarding; minors
37	McLaren, 2016	Official investigative report	International/state-supported doping	System-level governance failure
38	PHR & UCT, 2002	Professional guidelines/report	Occupational, military, detention health	Dual loyalty beyond sport
39	Sharkiya, 2023	Rapid review	Older patients, general healthcare	Communication–outcome links
40	Nwosu et al., 2023	Empirical — quantitative (survey)	Healthcare consumers	Trust as mediator of communication effects
41	Richards, 2022	Conceptual overview (encyclopedia entry)	General healthcare	Trust in physician: overview
42	Mann et al., 2007	Empirical — quantitative (survey)	US sports medicine physicians	Psychosocial issues in athlete care
43	Légaré et al., 2018	Systematic review (Cochrane)	Healthcare professionals	Effectiveness of SDM interventions
44	Li et al., 2020	Clinical commentary	Athletes with inherited cardiac disorders	SDM in clinically complex participation decisions
45	Nelis et al., 2024	Empirical — quantitative (survey)	Healthcare professionals working with athletes	SDM attitudes and practice
46	Anderson, 2007	Ethical analysis	Athletes; sports physicians	Risk-taking; consent; voluntariness
47	Schneider et al., 2019	Empirical — quantitative (survey)	German competitive athletes	Playing hurt
48	Diehl et al., 2019	Empirical — quantitative (survey)	German adolescent elite athletes	Playing hurt; joint pain
49	Bosco et al., 2012	Ethical analysis	Sports medicine	Ethics overview; confidentiality
50	Devitt & McCarthy, 2010	Ethical analysis (case-based)	Team doctors	Dual-loyalty dilemmas
51	Lacy et al., 2020a	Empirical — qualitative (interviews)	US collegiate athletic trainers	External pressures on clinical decisions

Ref.	Source	Publication type / evidence class	Setting and population	Contribution to framework domain(s)
52	Lacy et al., 2020b	Empirical — qualitative (interviews)	US collegiate athletic trainers	Organizational conflict in clinical decisions
53	Rothstein, 2016	Ethical/legal analysis	NFL (professional football, USA)	Critique of structural COI remedies
54	Duvernay-Tardif, 2016	Commentary (player-physician perspective)	NFL (professional football, USA)	Physician standards; relational safeguards
55	Partridge, 2014	Ethical analysis	Concussion management	Conflicts of interest
56	Herring et al., 2024	Professional consensus statement	Team physicians (USA)	Musculoskeletal assessment; return-to-play
57	Tenforde & Fredericson, 2015	Ethics case commentary	Sports medicine	Return-to-play ethics
58	Haley & Zhou, 2021	Narrative review	Team physicians	Return-to-play considerations
59	Reardon et al., 2019	International consensus statement	Elite athletes	Mental health; sensitive information
60	Bernstein et al., 2004	Ethical analysis	Sports medicine	Normative ethics
61	Peer, 2017	Conceptual analysis	Athletic training/sports medicine	Social contract; professional ethics
62	Schwarz, 2009	Illustrative case material (journalism)	NFL (professional football, USA)	Concussion recognition case
63	Kirk et al., 2013	Illustrative case material (documentary)	NFL (professional football, USA)	Concussion crisis case
64	USADA, 2012	Official investigative report	Professional cycling	Doping and medical governance failure
